# Correlates of protection and determinants of SARS-CoV-2 breakthrough infections 1 year after third dose vaccination

**DOI:** 10.1186/s12916-024-03304-3

**Published:** 2024-03-08

**Authors:** Carla Martín Pérez, Ruth Aguilar, Alfons Jiménez, Gemma Salmerón, Mar Canyelles, Rocío Rubio, Marta Vidal, Inocencia Cuamba, Diana Barrios, Natalia Díaz, Rebeca Santano, Pau Serra, Pere Santamaria, Luis Izquierdo, Antoni Trilla, Anna Vilella, Sonia Barroso, Marta Tortajada, Alberto L. García-Basteiro, Gemma Moncunill, Carlota Dobaño

**Affiliations:** 1 ISGlobal, Hospital Clínic, Universitat de Barcelona, Barcelona, 08036 Spain; 2 CIBER de Epidemiología y Salud Pública (CIBERESP), Barcelona, 08036 Spain; 3 Occupational Health Department, Hospital Clínic, Universitat de Barcelona, Barcelona, 08036 Spain; 4 Centro de Investigação Em Saúde de Manhiça, Maputo, CP 1929 Mozambique; 5 CIBER de Enfermedades Infecciosas (CIBERINFEC), Barcelona, 08036 Spain; 6 Institut d’Investigacions Biomèdiques August Pi Sunyer (IDIBAPS), Barcelona, 08036 Spain; 7 Department of Microbiology, Immunology and Infectious Diseases, Snyder Institute for Chronic Diseases, Cumming School of Medicine, University of Calgary, Calgary, AB T2N 1N4 Canada; 8 Department of Preventive Medicine and Epidemiology, Hospital Clinic, Universitat de Barcelona, Barcelona, 08036 Spain; 9 International Health Department, Hospital Clínic, Universitat de Barcelona, Barcelona, 08036 Spain

**Keywords:** SARS-CoV-2, Correlates of protection, Antibody response, Breakthrough infections, Omicron

## Abstract

**Background:**

The emergence of new SARS-CoV-2 variants and the waning of immunity raise concerns about vaccine effectiveness and protection against COVID-19. While antibody response has been shown to correlate with the risk of infection with the original variant and earlier variants of concern, the effectiveness of antibody-mediated protection against Omicron and the factors associated with protection remain uncertain.

**Methods:**

We evaluated antibody responses to SARS-CoV-2 spike (S) and nucleocapsid (N) antigens from Wuhan and variants of concern by Luminex and their role in preventing breakthrough infections 1 year after a third dose of mRNA vaccination, in a cohort of health care workers followed since the pandemic onset in Spain (*N* = 393). Data were analyzed in relation to COVID-19 history, demographic factors, comorbidities, vaccine doses, brand, and adverse events.

**Results:**

Higher levels of anti-S IgG and IgA to Wuhan, Delta, and Omicron were associated with protection against vaccine breakthroughs (IgG against Omicron S antigen HR, 0.06, 95%CI, 0.26–0.01). Previous SARS-CoV-2 infection was positively associated with antibody levels and protection against breakthroughs, and a longer time since last infection was associated with lower protection. In addition, priming with BNT162b2 followed by mRNA-1273 booster was associated with higher antibody responses than homologous mRNA-1273 vaccination.

**Conclusions:**

Data show that IgG and IgA induced by vaccines against the original strain or by hybrid immunization are valid correlates of protection against Omicron BA.1 despite immune escape and support the benefits of heterologous vaccination regimens to enhance antibodies and the prioritization of booster vaccination in individuals without recent infections.

**Supplementary Information:**

The online version contains supplementary material available at 10.1186/s12916-024-03304-3.

## Background

Coronavirus disease 2019 (COVID-19) caused by SARS-CoV-2 has posed significant challenges to global health. Even though the pandemic is deemed to be under control, it continues to present significant challenges to public health, particularly for vulnerable populations. As the virus continues to evolve, the emergence of new variants, such as the Omicron lineages (B.1.1.529), coupled to the decay with time of antiviral antibodies induced by natural infection or vaccination, led to a significant increase of COVID-19 cases, even among populations with high vaccination rates, thus raising concerns regarding vaccine effectiveness and immune protection [1, 2]. Although the administration of booster mRNA vaccines has proven effective in preventing severe cases of COVID-19 caused by Omicron [3], their ability to protect against infection seems limited [4–6].

Antibody response has been shown to correlate with the risk of infection with the ancestral virus and the earlier variants of concern [7–10]. Moreover, recent studies indicate that IgG and IgA antibody levels are associated with protection against Omicron infection [11–13]; however, the effectiveness of antibody-mediated protection against Omicron and the factors associated with protection against this infection variant are still not well defined. Hybrid immunity (combination of natural immunity and vaccine-induced immunity) has been reported to increase the magnitude and breadth of the immune response, with some studies also showing an association with protection against Omicron infection [13, 14]. Additionally, SARS-CoV-2 infection before or after vaccination has been reported to increase neutralizing antibody response against Omicron [15], and previous infection has also been shown to associate with protection against Omicron infection [11, 14, 15]. Nevertheless, the impact of the timing of infection and vaccination is not well established [15, 16]. This is of utmost importance in the current context of COVID-19, with different vaccine regimes and types and with a high proportion of the population exposed to immune-escaping variants in order to inform public health strategies and optimize individual protection.

Since the start of the pandemic, immuno-epidemiological studies in healthcare worker (HCW) cohorts have provided key information on the onset and evolution of antibody responses to SARS-CoV-2 infection and vaccination. In March 2020, we established a cohort of HCW at Hospital Clinic in Barcelona, Spain, that provided the first world estimates of SARS-CoV-2 seroprevalence [17] in this high-risk population, and its longitudinal follow-up allowed us to define the impact of preexisting antibodies to other coronaviruses on COVID-19 immune response and the kinetics of the antibodies against infection, as well as their determinants over time and early after primary vaccination [18, 19].

Here, we performed additional investigations on this well-characterized HCW cohort over a ≈ 3-year follow-up period to assess (i) the impact of prior SARS-CoV-2 infection, booster immunization (third dose) with COVID-19 mRNA vaccines, and clinical and demographic factors, on antibody levels 2 years after the onset of the pandemic; (ii) the correlation of those antibody responses with protection against COVID-19 during a 10-month period of predominant Omicron transmission; and (iii) the effect of the aforementioned variables on vaccine breakthrough infections.

## Methods

### Study design and setting

A cohort of 578 HCWs providing direct or indirect patient care at Hospital Clínic in Barcelona randomly selected was followed up for ≈3 years since the beginning of the pandemic in Spain. Questionnaires and sample collection were done over 7 visits: M0 (baseline, month 0, 28 March–9 April 2020, *n* = 578), M1 (month 1, 27 April–6 May 2020, *n* = 566), M3 (month 3, 28 July–6 August 2020) (*n* = 70), M6 (month 6, 29 September–20 October 2020, *n* = 507), M9 (month 9, 19 January–5 Feb 2021, *n* = 132), M12 (month 12, 12 February–30 April 2021, *n* = 414), and M24 (month 24, 14 March–25 April 2022, *n* = 393) (32% of initial participants were lost to follow-up). At M3 and M9, only participants with previous evidence of infection were invited to participate in the survey. Up to 17 January 2023 (end of the follow-up period), 114 participants have had the 4 vaccine dose (vaccination period ranged from October 2022 to January 2023).

Participant data were collected over the 7 study visits using a standardized electronic questionnaire through REDCap version 13.8.1 as previously described. Additionally, data on confirmed SARS-CoV-2 infections and COVID-19 symptoms, vaccination type, and dates and adverse effects up to 10 months after the M24 visit were collected at the Occupational Health and Preventive Medicine and Epidemiology departments at the Hospital Clinic. Molecular data regarding the SARS-CoV-2 variants was not collected routinely in the hospital. During the M0 and M1 visits, active detection of infection by real-time reverse transcription-polymerase chain reaction (rRT-PCR) was conducted. For subsequent visits, rRT-PCR or AgRDT (Antigen Rapid Diagnostic test) were performed whenever participants exhibited symptoms or had contact with an infected individual. In addition, individuals with a positive serology before vaccination or a positive N serology at any timepoint were also considered infected. When previous infection was detected by serology, the timepoint interval when this infection occurred was defined as the interval when a participant seroconverted for any of the antigens (for timepoints before vaccination) or for N antigen (for any timepoint).

### Quantification of antibodies to SARS-CoV-2

IgA, IgG, and IgM levels (median fluorescence intensity, MFI) to S and N SARS-CoV-2 antigens were measured by Luminex-based assays (quantitative suspension array technology) in multiplex as previously described [19, 20]. The panel of antigens included the S full length, the subregion S1, and the receptor-binding domain (RBD) of S1, expressed in lentiviruses as described [21], as well as the subregion S2 (SinoBiological) and the nucleocapsid (N) protein (expressed in *E. coli*), all from the Wuhan strain. As variants of concern, the full-length S proteins from Delta and Omicron BA.1 (SinoBiological) were also included. Plasma samples were tested at 1:500 dilution for the 3 isotypes and additionally at 1:5000 for IgG to avoid saturated anti-S levels in the vaccinated participants. Optimal testing dilutions were previously assessed and samples were within the quantitative range of the assay. The investigators conducted the assays blinded.

### Statistical analysis

MFI values were log10-transformed for analysis. Seropositivity was calculated with the values measured at 1:500 plasma dilution. The cutoffs for each isotype and antigen were calculated as 10 to the mean plus 3 standard deviations (SD) of the log10-transformed MFI from 128 pre-pandemic (negative) controls. Positive serology was defined as being positive for IgG, IgA, and/or IgM to any of the tested antigens, and serology was considered undetermined when the MFI levels for a specific isotype-antigen fell between the positivity threshold and 10 to the mean plus 4.5 SD of the log10-transformed MFIs of the negative controls, provided that no other isotype-antigen combination exceeded the positivity threshold.

The differences between groups at M24 were measured using a two-tailed Wilcoxon Rank- Sum test. The differences between timepoints within a group were measured with a Wilcoxon signed-rank test. MFI divided by the cutoff value was used when two timepoints were compared to address variations in MFI measures between timepoints. To account for multiple testing, both tests were adjusted using the Benjamini–Hochberg method [22]. All tests were performed two-sided. The strength of correlations between antibody levels was evaluated by Pearson’s correlation test.

Univariable and multivariable linear regression models were fitted to assess factors associated with antibody responses to SARS-CoV-2 at M24 among exposed, naive, and all individuals. Separate models were also fitted for individuals vaccinated with at least 2 doses and for those vaccinated with 3 doses. The regression coefficients (β) obtained from each model were converted into percentage values to facilitate interpretation. The transformed β value (%) was calculated using the formula ((10^β) − 1) × 100. This indicates the percentage difference in the dependent variable associated with a 1-unit increase in the corresponding independent variable (for continuous variables) or the percentage difference in the dependent variable between the reference group and the study group (for categorical variables). Causal assumptions for each of the analyses are reported in directed acyclic graphs (DAGs), which can be found in the supplementary material (Additional File 1: Fig. S15 - S18).

The relationship between antibody levels and risk of SARS-CoV-2 breakthrough infection was modeled using logistic generalized additive models (GAM) with cubic splines, for examining the non-linear relationship between continuous predictor and the response.

To investigate the effect of antibody levels on the risk of SARS-CoV-2 breakthrough infection, we conducted a survival analysis using both Kaplan–Meier analysis and multivariate Cox regression modeling. Individuals with at least two doses were considered at risk of infection from M24 visit until the occurrence of the first reported episode of SARS-CoV-2 breakthrough infection, the receipt of the fourth dose of the vaccine, or the last day of study follow-up established for the analysis reported here (17 January 2023). Causal assumptions for each analysis are reported in DAGs, which can be found in the supplementary material (Additional File 1: Fig. S19 - S20).

To identify the most influential antibody predictors of breakthrough infection, we employed a Cox regression with LASSO regularization. This method helps to address collinearity by penalizing and shrinking the coefficients of highly correlated predictors, resulting in a more robust model. Prior to analysis, each antibody variable was log_10_-transformed and scaled to have mean 0 and standard deviation 1. One thousand replicates of nested fivefold cross validation using *λ* equal to lambda.min (the value which minimized leave-one-out cross validation misclassification) were performed to assess model stability. For final regression, lambda.min and lambda.1se values were obtained using leave-one-out cross-validation, with models shown for the complete path of *λ* values.

Hierarchical clustering was performed using the complete linkage method on the Euclidean distances of the normalized variables. Heatmap was plotted using the R package pheatmap (v1.0.12).

Missing data were handled by excluding cases with incomplete information. The sample sizes for each analysis are indicated in the corresponding table and figure legends. Adjusted *p*-values lower than 0.05 were considered statistically significant. We performed the statistical analysis in R version 4.2.2.

## Results

### Cohort and study design

**Fig. 1:**
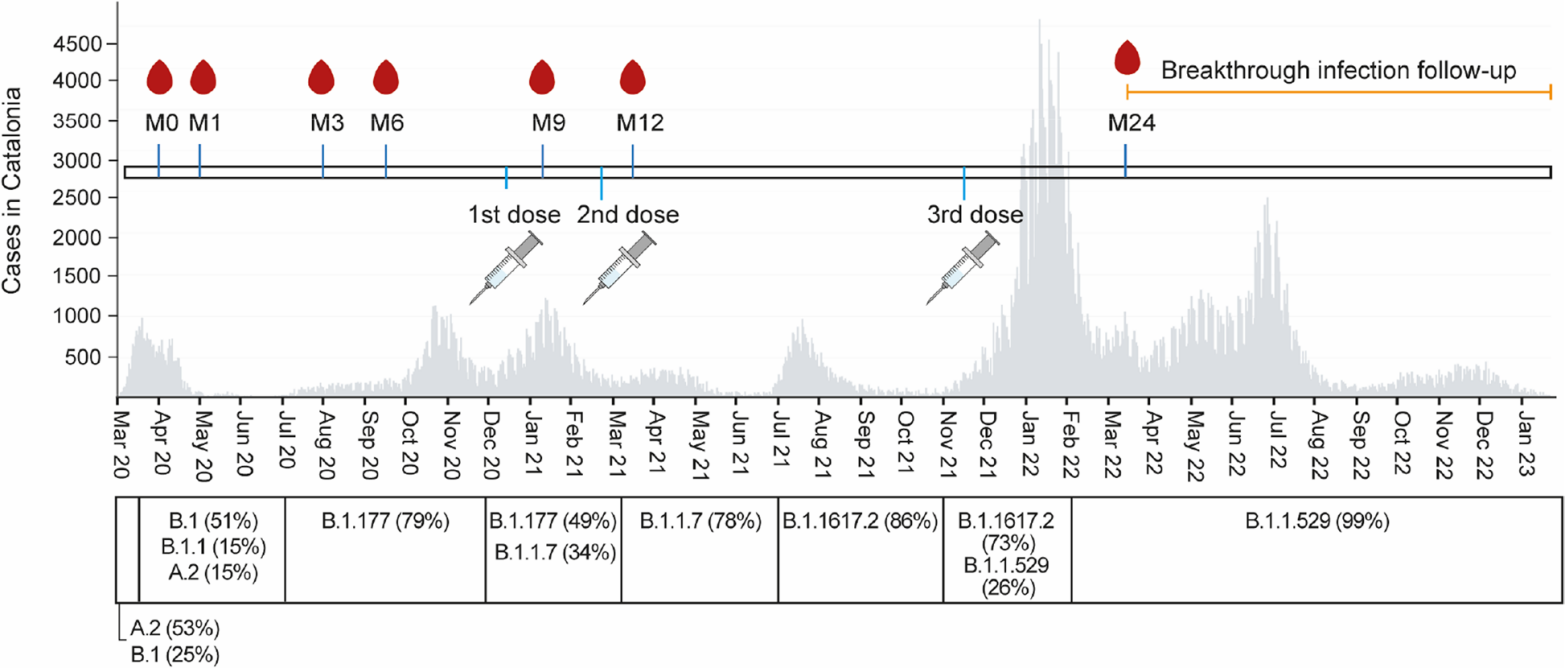
SeroCoV/SeroCoVac study sample collection and vaccination timepoints, SARS-CoV-2 cases in Catalonia, and main SARS-CoV-2 lineages circulating in Spain over the 3-year study period. The grey plot represents the number of SARS-CoV-2 cases at a given time in Catalonia according to the official data available from the Spanish National Epidemiological Surveillance Network (RENAVE, https://cnecovid.isciii.es/covid19 (accessed on 12 July 2023)). The bottom timeline depicts the main SARS-CoV-2 lineages circulating in Spain at those time intervals (shown as percentage). The orange line represents the follow-up period (10 months) for breakthrough infections in this study. M, month

Out of the 578 HCW recruited at the beginning of the pandemic and visited at 6 time points over 2020–2021 [17, 19, 23, 24], 393 participants came to the month (M)24 visit between 14 March and 25 April 2022 (32.0% lost to follow-up) (Fig. 1). At M24, most of the participants (*n* = 370) had received the primary series of vaccination (67.0% BNT162b2 and 33.0% mRNA-1273) and 287 received the 1st booster dose (0.7% BNT162b2 and 99.3% mRNA-1273) (Table 1). The mean time since the 2nd vaccine dose was 381 days (SD 90.7), while the mean time since the 3rd dose (booster) was of 115 days (SD 15.8). At M24, 99.7% of the individuals (*n* = 392) were seropositive. Up to M24, 61.8% of the participants (*n* = 243) had been previously infected by SARS-CoV-2 according to rRT-PCR (77.0%) or only serology data (23.0%), and 38.1% (*n* = 150) had no evidence of infection. The mean time since last infection was 172 days (SD 105.6). Of those without evidence of previous infection, 1 had one vaccine dose (0.7%), 20 (13.3%) had two vaccine doses, and 129 (86.0%) had three doses, while among those with infection, 11 (4.5%) were not vaccinated, 11 (4.5%) had 1 dose, 63 (25.9%) had 2 doses, and 158 (65.0%) had 3 doses. Among those with 3 doses (*n* = 287), 69 (24.0%) had evidence of infection previous to primary vaccination, 15 (5.2%) after primary vaccination but before booster, and 71 (24.7%) post-booster. Most of the study participants were females (73.0%) and had a mean age of 46.7 (SD 11.2) years (Table 1). Around 29.7% had underlying comorbidities, and among those, 78.6% were under chronic medication (Table 1). Among the 370 participants vaccinated with at least two doses, 109 (29.4%) had vaccine breakthroughs post-M24 detected by rRT-PCR from 14 March 2022 (M24) up to 17 January 2023 (Table 1). Seropositivity of study participants at M24 is described on Additional File 1: Table S1.

**Table 1:** Characteristics of study participants at M24

		*N* or mean + SD
Sex	Male	106
	Female	287
Professional category	Nurse/auxiliary/stretcher-bearer	192
	Physician	93
	Lab technician	14
	Admin officers/other	94
Age		46.7 ± 11.2
Comorbidities	Yes	117
	No	276
Comorbidity category^a^	Cardiovascular	41
	Respiratory disease	33
	Endocrine	33
	Gastrointestinal	12
	Neurological	10
	Dermatological	10
	Immunosuppression	6
	Hematological	4
	Cancer	4
	Liver	3
	Gynecological	3
	Mental health	3
	Immunological	2
	Other^b^	111
Chronic medication^a^	Yes	92
	No	25
Smoker	Yes	79
	No	313
	NA	1
No. of people in the household		2.8 ± 1.3
Involved in clinical care	Yes	285
	No	108
1st dose vaccination	Pfizer	251
	Moderna	131
2nd dose vaccination	Pfizer	248
	Moderna	122
3rd dose vaccination	Pfizer	2
	Moderna	285
Number of doses received	0	11
	1	12
	2	83
	3	287
Breakthrough infection (post M24)	Yes	109
Among vaccinated with 2 or 3 doses	No	261

^a^Among those with comorbidities

^b^Includes age-related bone and muscular disorders as well as renal and neuromuscular disorders

### Comparison of antibody levels following hybrid immunity or vaccination alone

**Fig. 2:**
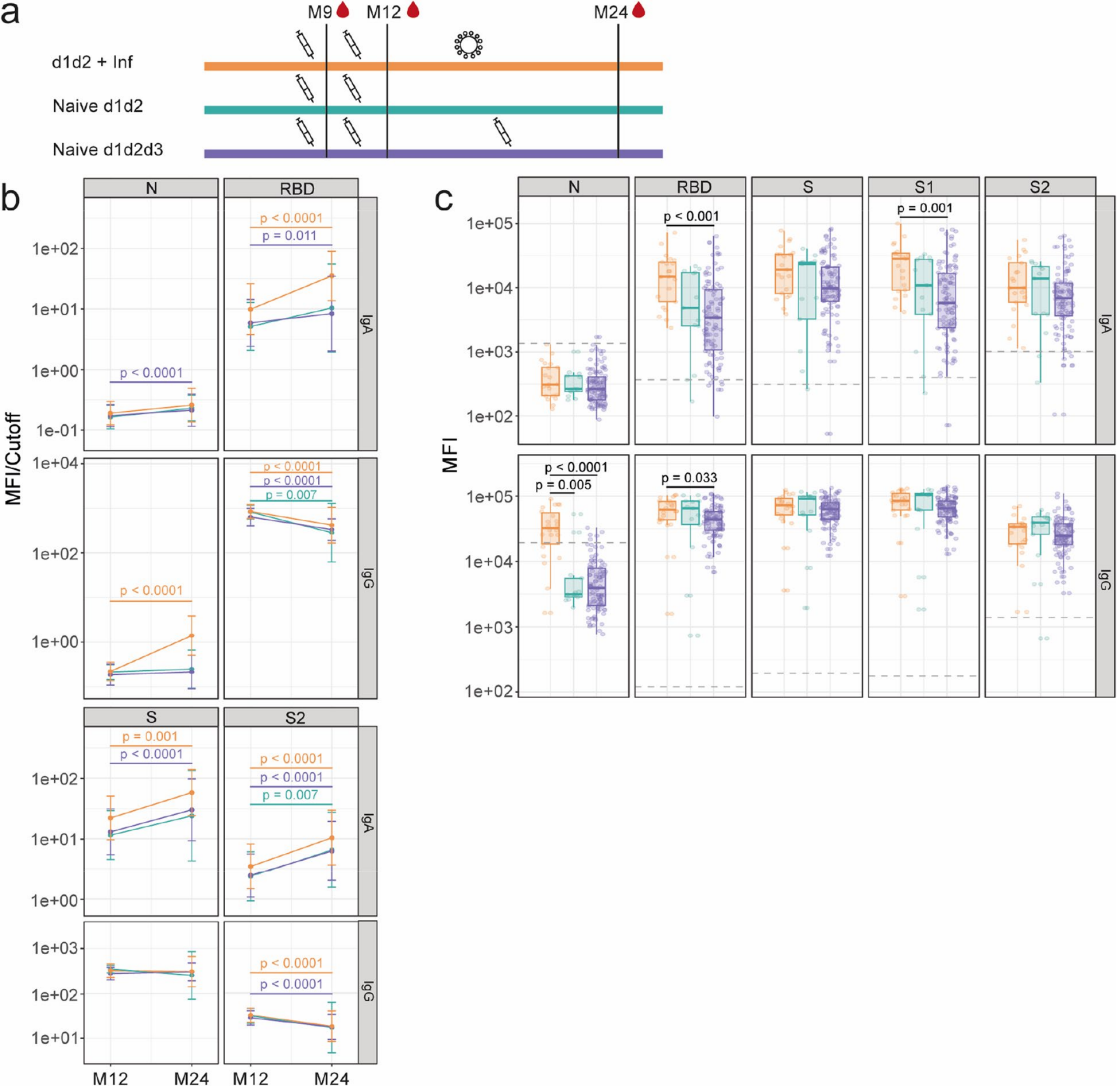
Evolution and comparison of antibody levels following hybrid immunity or vaccination alone. Plots show IgA and IgG levels against the receptor-binding domain (RBD) of the SARS-CoV-2 Spike glycoprotein (S), S, its subunits S1 and S2, and against the nucleocapsid (N). **a** Scheme illustrating the order and approximate time scale of vaccination and infection for each group. The syringe indicates a vaccine dose, the virus particle indicates an infection with SARS-CoV-2, and the blood droplet indicates plasma collection for antibody measurement. **b** Antibody levels (MFI/cutoff) at M12 and M24 time points in individuals vaccinated with 2 doses followed by an infection (*n* = 22), 2 doses without infection (*n* = 13), and 3 doses without infection (*n* = 129). The data shown are geometric mean in each group ± geometric SD. Statistical significance was tested by Wilcoxon signed-rank test and adjusted for multiple comparisons using Benjamini–Hochberg method. **c** Comparison of antibody levels (MFI) at M24 according to exposure. The center line on each box corresponds to the median MFI, the lower and upper hinges correspond to the first and third quartiles, and the whiskers extend from the hinge to the highest or lowest value within 1·5 × IQR of the respective hinge. Grey dashed line depicts the positivity cutoff for the given antibody. Statistical significance was tested by Wilcoxon rank-sum test and adjusted for multiple comparisons using Benjamini–Hochberg method. S1 antigen was only measured at M24

Infection or booster vaccination after dose 2 (between M12 and M24) (Fig. 2a, b) were associated with an increase in IgA levels against the receptor-binding domain (RBD), spike (S), and S2 antigens between the two study visits, whereas naive individuals vaccinated with 2 doses had a non-significant increase for RBD or S antigens (Fig. 2b). However, IgG levels waned or were maintained from M12 to M24 regardless of infection or booster vaccination after dose 2 (Fig. 2b). At M24, infection after dose 2 was associated with higher levels of IgA to S1 and RBD, and IgG to RBD, than booster vaccination (Fig. 2c). This was also the case for IgG and IgA against Omicron S (Additional File 1: Fig. S1). Regarding IgM, levels of anti-RBD and anti-S antibodies decreased from M12 to M24 in all three groups, while levels of anti-nucleocapsid (N) antibodies increased in the three groups, and levels of anti-S2 antibodies only decreased significantly for those naive vaccinated with 3 doses (Additional File 1: Fig. S2). In addition, at M24, anti-S1 IgM levels were higher in individuals with two doses and infection than in those naive vaccinated with 2 doses. These findings indicate that infection may induce a higher antibody response than vaccination for these antibodies.

### Impact of timing of infection relative to vaccine doses on antibody levels

Post-booster infection resulted in increased or stable IgG levels from M12 to M24 (Additional File 1: Fig. S3a, b). Conversely, pre-primary vaccination infections and post-primary pre-booster infections resulted in stable or decreased antibody levels from M12 to M24 (Additional File 1: Fig. S3b). Moreover, post-booster infection was positively associated with an increase in IgA antibody levels from M12 to M24, particularly for antibodies targeting N and RBD. In naïve vaccinated individuals, there was also an increase in IgA levels from M12 to M24 due to the booster dose.

At M24, post-booster infection was associated with elevated levels of both IgA and IgG to N and S antigens (Additional File 1: Fig. S3a, c). Compared to pre-primary infection, post-booster infection was associated with higher antibody levels to RBD, S, S1, and N antigens. Notably, post-booster infection led also to higher IgG levels compared to post-primary pre-booster infection (Additional File 1: Fig. S3c). Regarding IgM, there were some differences in magnitude, but the vast majority of participants had levels below the seropositivity cutoff (Additional File 1: Fig. S4). Post-booster infection was also associated with higher levels of IgA and IgG to S from Delta and Omicron variants compared to the other 3 groups, except for IgA against S from Delta variant, which was only higher than those naive and infected pre-primary vaccination. (Additional File 1: Fig. S5).

**Fig. 3:**
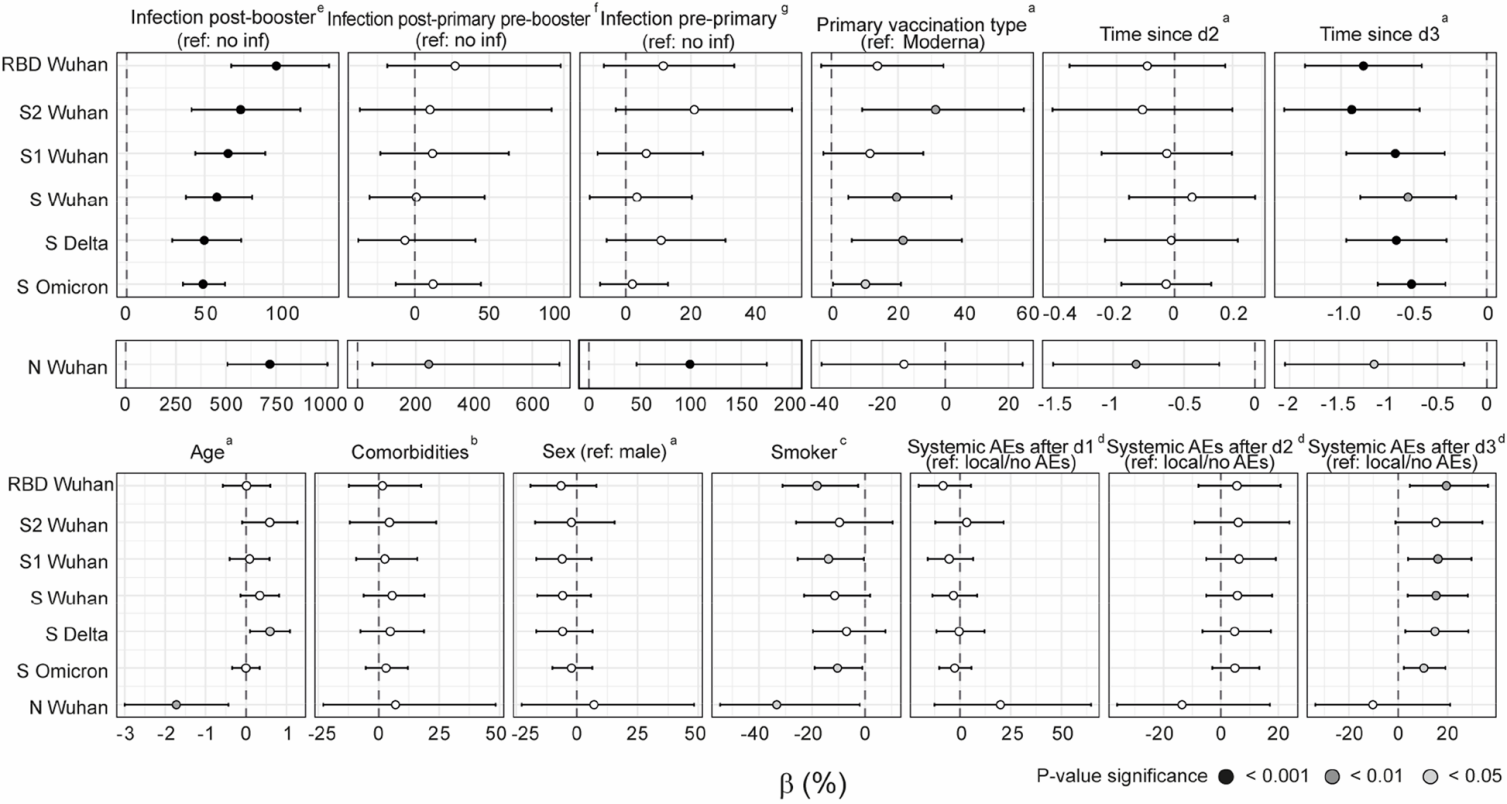
Linear regression analysis of the association of several factors with M24 IgG antibody levels in individuals vaccinated with 3 doses. The estimates (dots) and 95% CI were calculated using linear regression models. Beta (*β*) and CI values have been transformed to a percentage for an easier interpretation. The color of the dots represents the *p* value after adjustment for multiple testing by Benjamini-Hochberg, where black represents < 0.001, dark grey < 0.01, light grey < 0.05, and white non-significant. “^a^” indicates the following: non-adjusted variable. *n* = 287. “^b^” indicates the following: covariates for adjustment included age, sex, and smoking. *N* = 287. “^c^” indicates the following: covariates for adjustment included age and sex. *n* = 287. “^d^” indicates the following: covariates for adjustment included age, comorbidities, chronic medication, sex, smoking, and primary vaccination type. *n* = 287. “^e^” indicates the following: covariates for adjustment included age, comorbidities, chronic medication, infection post-primary pre-booster vaccination, infection pre-primary vaccination, sex, smoking, primary vaccine type, AEs after dose 1, AEs after dose 2, and AEs after dose 3. *n* = 245. Interaction terms were included between post-booster infection and infection pre-primary vaccination and infection post-primary vaccination pre-booster. “^f^” indicates the following: covariates for adjustment included age, comorbidities, chronic medication, infection pre-primary vaccination, sex, smoking, primary vaccine type, AEs after dose 1, and AEs after dose 2. Interaction terms were included between infection post-primary vaccination pre-booster and infection pre-primary vaccination and post-booster vaccination. *n* = 245. “^g^” indicates the following: covariates for adjustment included age, comorbidities, chronic medication, sex, smoking, and primary vaccine type. Interaction terms were included between infection pre-primary vaccination and post-booster vaccination and post-primary vaccination pre-booster. *n* = 245

Among infected participants with 3 doses, a longer time since the last infection was associated with lower IgG levels for all antigens and variants assessed in non-adjusted linear models (Additional File 1: Fig. S6). Along these lines, post-booster infections were strongly associated with higher antibody levels, particularly to N, in models adjusted for age, comorbidities, chronic medication, infection post-primary pre-booster vaccination, infection pre-primary vaccination, sex, smoking, primary vaccine type, and adverse events (AEs) after doses 1, 2, and 3 (Fig. 3). In contrast, infections occurring before primary vaccination and post-primary pre-booster vaccination were only associated with higher anti-N but not anti-S antigens antibody levels (in models adjusted for age, comorbidities, chronic medication, sex, smoking, and primary vaccine type, or age, comorbidities, chronic medication, infection pre-primary vaccination, sex, smoking, primary vaccine type, AEs after doses 1 and 2, respectively). These findings indicate the timing of infection greatly influences the impact of previous infection on antibody levels against Wuhan, Delta, and Omicron variants.

### Analysis of other factors associated with antibody levels

Having 3 compared to 2 doses was positively associated with IgG levels against S, S1, RBD, and N from Wuhan and S from Delta variant in multivariable analysis (Additional File 1: Table S2). However, no significant association was detected with IgG against S from Omicron. Additionally, having 3 doses was negatively associated with anti-N IgG levels, reflecting lower incidence of infection in those vaccinated with 3 doses.

Primary vaccination with BNT162b2 followed by mRNA-1273 booster was associated with an increase in IgA levels to all antigens from M12 to M24, while those vaccinated with mRNA-1273 in primary and booster vaccination showed an increase only for antibodies to N and S2 (Additional File 1: Fig. S7a). IgG levels waned for RBD and S2 regardless of type of vaccination, while IgG levels to S increased for those primary vaccinated with BNT162b2. At M24, primary vaccination with BNT162b2 followed by mRNA-1273 booster was associated with higher levels of both IgA and IgG antibodies to S2 compared to 3 doses of mRNA-1273 (Additional File 1: Fig. S7b) and higher anti-S IgA levels. For Omicron and Delta S, levels of IgA to both variants and IgG to Delta variant were also higher in the heterologous vaccinees at M24 (Additional File 1: Fig. S8). In non-adjusted linear models, primary vaccination with BNT162b2 followed by mRNA-1273, compared to homologous vaccination with mRNA-1273, was associated with higher levels of IgG to S antigens for all variants (for all participants and for those infected) (Fig. 3, Additional File 1: Fig. S6).

Systemic AEs compared to local or no AEs after third dose were associated with increased levels of IgGs to S antigens from the three variants (for all of participants and for those naive) (Fig. 3, Additional File 1: Fig. S9) in models adjusted for age, comorbidities, chronic medication, sex, smoking, and primary vaccination type. Systemic AEs compared to local or no AEs after a second dose were only linked to increased levels of anti-S IgG in naive individuals with 3 doses (Additional File 1: Fig. S9), while systemic AEs after first dose were negatively associated with IgG levels to Wuhan S antigens in infected individuals (Fig. 3, Additional File 1: Fig. S6) in models adjusted for age, comorbidities, chronic medication, sex, smoking, and primary vaccination type. Age was negatively associated with anti-N IgG levels and positively associated with IgG to S from Delta variant among participants with 3 doses in non-adjusted linear models (Fig. 3). Smoking also exhibited a negative association with IgG levels to S1, RBD, and N from Wuhan variant and S from Omicron variant, among participants with 3 doses and with IgGs to all S antigens in infected individuals with 3 doses in models adjusted by age and sex (Fig. 3, Additional File 1: Fig. S6).

### Effect of antibody levels on the risk of SARS-CoV-2 breakthrough infection

**Fig. 4:**
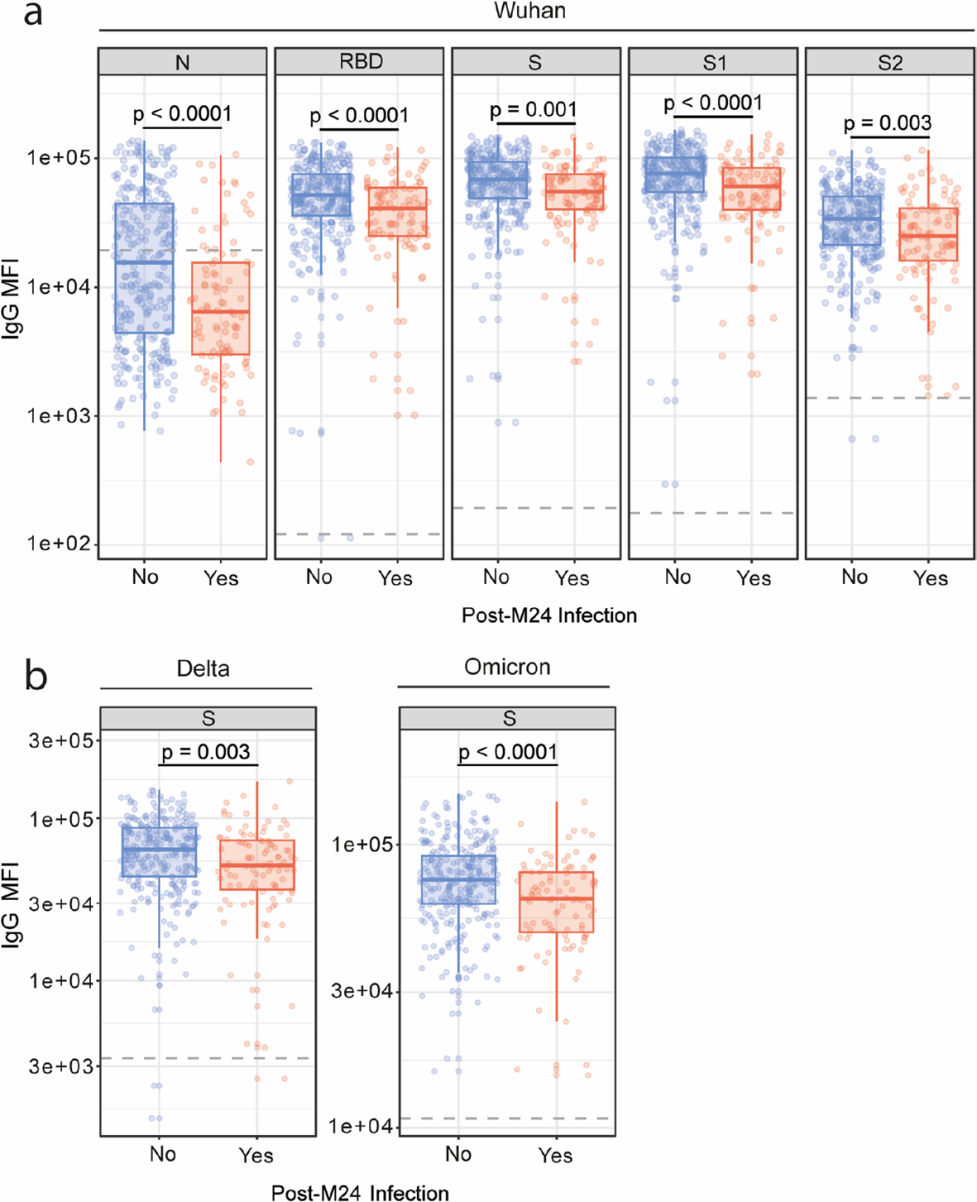
Association of IgG levels at M24 with post-M24 breakthrough infections. **a** IgG levels (MFI) at month (M)24 against the receptor-binding domain (RBD) of Wuhan SARS-CoV-2 Spike glycoprotein (S), S, its subunits S1 and S2, and against the nucleocapsid (N), in breakthrough infected (*n* = 95) and non-breakthrough infected (*n* = 274) individuals. **b** IgG levels (MFI) at M24 against Delta and Omicron BA.1 S. The center line on each box corresponds to the median MFI, the lower and upper hinges correspond to the first and third quartiles, and the whiskers extend from the hinge to the highest or lowest value within 1·5 × IQR of the respective hinge. Statistical significance was tested by Wilcoxon rank-sum test and adjusted for multiple comparisons using Benjamini–Hochberg method

IgG levels to all antigens and variants tested at M24 were lower in those individuals with a post-M24 infection compared to the non-infected. (Fig. 4). This was also the case for IgA to S antigens (Additional File 1: Fig. S10) but not IgM levels, which were not correlated with post-M24 infection (data not shown).

**Fig. 5:**
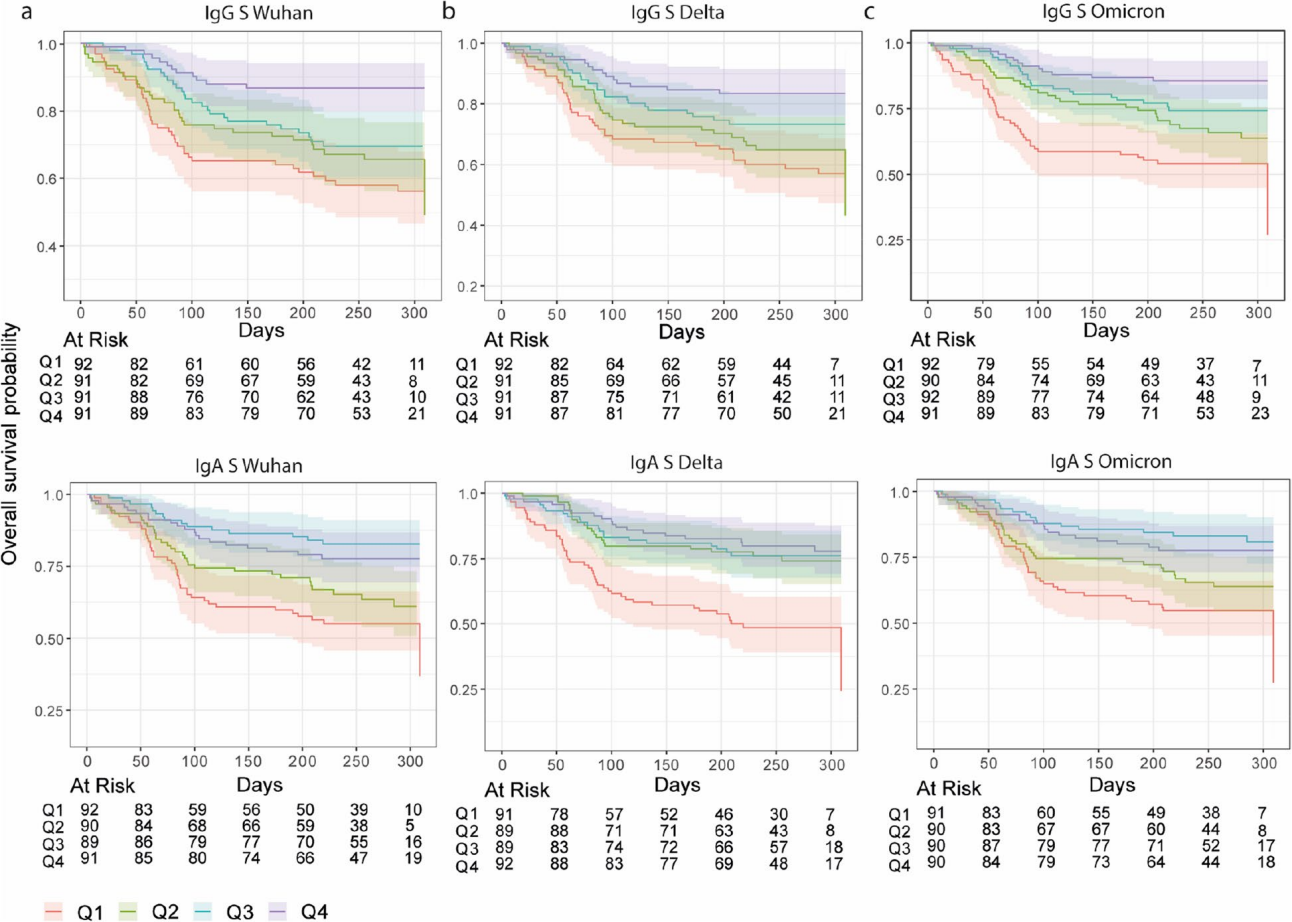
Kaplan–Meier survival curves of risk of breakthrough infection by quartiles of anti-Spike (S) IgG and IgA levels at month 24. Shaded areas represent the 95% confidence intervals

**Fig. 6:**
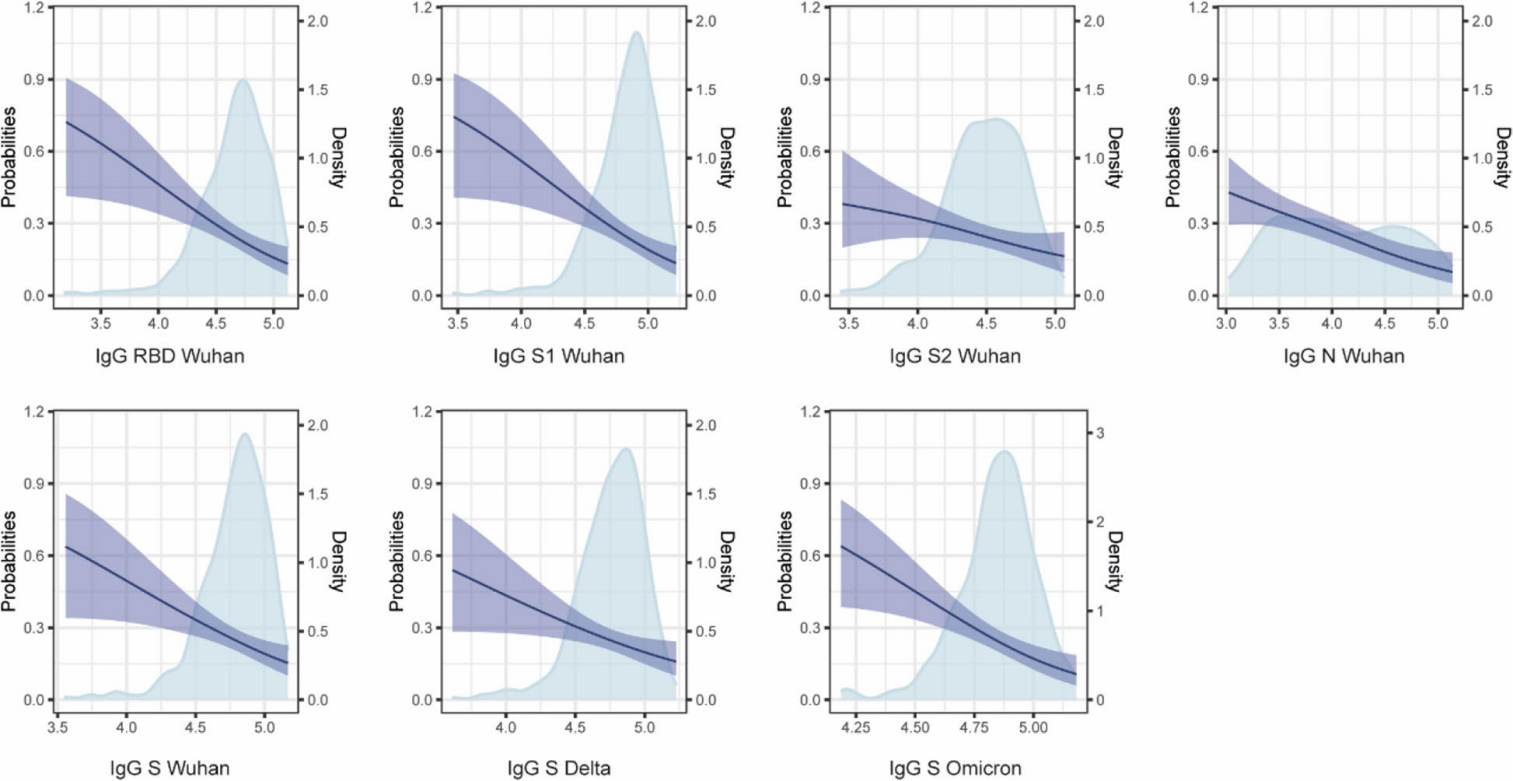
Predicted risk of breakthrough infection as a function of IgG antibody levels measured at month (M)24. Blue shaded areas represent the density distribution for each antibody. Purple lines show the risk of infection. Purple shaded areas represent the 95% confidence intervals for the risk

Stratifying anti-S antibody levels in quartiles, we found that the risk of breakthrough infections for the lowest and highest quartiles began to diverge from the risk for highest quartile after around 50 days of follow-up for IgG and around 80 days for IgA (Fig. 5). Along these lines, when we examined the relationship between risk of breakthrough infection and antibody levels using a GAM model, the risk significantly decreased with increasing levels of anti-S and anti-N IgG and IgA antibodies (*p* < 0.01) (Fig. 6, Additional File 1: Fig. S11).

Multivariable models (adjusted for age, comorbidities, chronic medication, sex, smoking, infection, time since last infection, vaccination with 3 doses (ref: 2 doses), and time since last dose) estimated that higher levels of IgA and IgG to S antigens (RBD, S, S1, S2) were associated with protection against breakthrough infection (Table 2, Additional File 1: Table S3). Hazard ratios associated with each tenfold increase in antibody levels was 0.25 (95% CI, 0.11–0.57) and 0.57 (95% CI, 0.38–0.86) for IgG and IgA against Wuhan S, respectively. IgG and IgA levels to both Omicron and Delta S variants were also found to be associated with protection: HR Omicron 0.06 (95% CI, 0.01–0.26) for IgG, 0.20 (95% CI, 0.07–0.56) for IgA, HR Delta 0.30 (95% CI, 0.14–0.66) for IgG, 0.61 (95% CI, 0.39–0.96) for IgA (Table 2, Additional File 1: Table S3).

**Table 2:** Summary of multivariable Cox regression models to assess the association of antibody levels with breakthrough infection in individuals vaccinated with two or three doses of mRNA vaccine

Isotype	Antigen	Variant	HR	Upper CI	Lower CI	*p*-value
IgA	N	Wuhan	0.73	1.65	0.32	0.4508
IgA	RBD	Wuhan	0.61	0.90	0.41	**0.0126**
IgA	S	Wuhan	0.57	0.86	0.38	**0.0068**
IgA	S1	Wuhan	0.63	0.94	0.43	**0.0220**
IgA	S2	Wuhan	0.68	1.05	0.44	0.0784
IgG	N	Wuhan	0.70	1.23	0.39	0.2158
IgG	RBD	Wuhan	0.27	0.54	0.14	**0.0002**
IgG	S	Wuhan	0.25	0.57	0.11	**0.0008**
IgG	S1	Wuhan	0.24	0.53	0.11	**0.0003**
IgG	S2	Wuhan	0.38	0.75	0.19	**0.0051**
IgA	S	Delta	0.61	0.96	0.39	**0.0334**
IgG	S	Delta	0.30	0.66	0.14	**0.0026**
IgA	S	Omicron	0.20	0.56	0.07	**0.0021**
IgG	S	Omicron	0.06	0.26	0.01	**0.0001**

Covariates for adjustment included age, comorbidities, chronic medication, sex, smoking, infection, time since last infection, vaccination with 3 doses (ref: 2 doses), time since last dose. *N* = 277. Among the 277 individuals included in the model, there were 75 breakthrough infections

*HR* hazard ratio

Altogether, these findings suggest that higher anti-S and anti-N IgG and IgA levels were associated with a reduced risk of a breakthrough infection during a period (2022) when Omicron was the dominant infecting variant in Spain.

### Analysis of factors associated with risk of breakthrough infection

Previous infection had a strong negative association with breakthrough infection (HR, 0.13; 95% CI, 0.06–0.29) in models adjusted for age, comorbidities, chronic medication, sex, smoking, and vaccination with 3 doses (ref: 2 doses) (Table 3, Additional File 1: Table S4). However, the analysis also revealed that the time since the last infection was negatively associated with the level of protection (HR associated to 30 days, 1.1, 95% CI, 1.03–1.20) (Table 3, Additional File 1: Table S4). In those participants vaccinated with 3 doses, previous infection following booster vaccination was also strongly associated with protection against breakthrough infection (HR, 0.043, 95% CI, 0.18–0.01). Nevertheless, infections occurring before primary vaccination and infections following primary vaccination but prior to booster vaccination were not associated with protection. These findings emphasize the importance of time since the last infection in the level of protection.

**Table 3:** Summary of Cox regression models to assess the association of clinicodemographic factors with breakthrough infection in individuals vaccinated with two or three doses of mRNA vaccine

Factor	HR	Upper CI	Lower CI	*p*-value
Age^a^	0.989	1.008	0.969	0.2562
Comorbidities^b^	0.865	1.346	0.556	0.5226
Infection (ref: no infection, at day 0)^c^	0.130	0.289	0.059	**< 0.0001**
Sex (ref: male)^a^	1.465	2.322	0.924	0.1044
Smoker^d^	0.768	1.261	0.468	0.2980
Time since last dose (days)^e^	1.001	1.004	0.997	0.6598
Time since last infection (days)^c^	1.003	1.006	1.001	**0.0014**
Vaccination with 3 doses (ref: 2 doses)^f^	1.546	7.720	0.309	0.5950

*HR* hazard ratio

^a^Non-adjusted variable. *N* = 369

^b^Covariates for adjustment included age, sex, and smoking. *N* = 368

^c^Covariates for adjustment included age, comorbidities, chronic medication, sex, smoking, and vaccination with 3 doses (ref: 2 doses). An interaction term was included between infection and time since last infection. *N* = 277

^d^Covariates for adjustment included age and sex. *N* = 368

^e^Covariates for adjustment included vaccination with 3 doses (ref: 2 doses). *N* = 369

^f^Time since dose 3 was included as an interaction. *N* = 369

When the impact of previous infection was analyzed using a model adjusted for antibody levels, which are mediators of the protective effect, the HR associated with previous infection was notably reduced (HR at day 0, 0.27, 95% CI, 0.75–0.1). Nevertheless, the results still indicate that previous infection confers a protective effect independent of antibody levels.

Furthermore, in non-adjusted models, vaccination with 3 doses as compared with 2 doses was not found to be associated with protection against breakthrough infection (HR 1.54, 95% CI, 7.7–0.31, non-significant) (Table 3). This was also the case for models adjusted for previous infection (HR 1.45, 95% CI, 2.7–0.77, non-significant).

### Correlates of protection and combined effect of antibody levels on protection against breakthrough infection

A strong degree of correlation was found within IgG and IgA levels to S antigens, while a moderate correlation was observed between IgG and IgA levels against S antigens, and IgG and IgA levels against N (Additional File 1: Fig. S12). Antibodies to N and S antigens were poorly correlated (Additional File 1: Fig. S12).

To identify the most influential antibody predictors of breakthrough infection while addressing their collinearity, we employed a LASSO penalized Cox regression model (Additional File 1: Fig. S13). This approach allowed us to effectively select the antibody variables that had the strongest association with the risk of breakthrough infection, while mitigating the impact of multicollinearity among the predictors. In the penalized regression analysis, which incorporated the levels of all 14 different antibodies as independent variables, only IgA to Omicron S and IgG to Wuhan N and Omicron S were selected (Additional File 1: Fig. S13). Those antibody variables consistently maintained a negative coefficient and were included in the model in all of the cross-validation rounds, reinforcing their robust association with breakthrough infection.

To further explore the relationship of antibody levels with subsequent breakthrough infection, and to find potential patterns (combined effect of antibodies) associated with breakthrough infection outcome, we conducted a hierarchical clustering analysis which included levels of IgG and IgA to Omicron S and IgG and IgA to N (Additional File 1: Fig. S14). We observed two distinct clusters (Additional File 1: Fig. S14), the first consisting predominantly of individuals who experienced breakthrough infections, while the second mainly comprised non-infected individuals. Within the predominantly infected cluster, most individuals had low levels of IgA to both S and N. Additionally, IgG levels to N were generally moderate to low, while IgG levels to S were relatively high for the majority of individuals in this cluster. In contrast, in the predominantly non-infected cluster, IgA levels to N were mostly low (except for a subset of participants), while IgA levels to S and IgG levels to N were notably higher for most individuals. Moreover, IgG levels to S were also higher in this cluster compared to the predominantly infected cluster. Notably, individuals who displayed high levels of all four antibodies had the lowest incidence of breakthrough infections. These findings suggest that a combination of elevated IgA and IgG against S and IgG against N may contribute to a reduced risk of breakthrough infections, where the association with elevated anti-N antibody levels may be a surrogate for additional immune responses related to recent infections.

To assess the individual contributions of IgG against S and N to the risk of breakthrough infection, we constructed an adjusted Cox regression model incorporating antibody levels of both IgG and IgA against N from Wuhan variant and against S from Omicron variant (Table 4) and additional covariates (age, comorbidities, chronic medication, sex, smoking, vaccination with 3 doses (ref: 2 doses), and type of primary vaccination). In this model, IgG to Omicron S (HR, 0.12, 95% CI, 0.027–0.50) and to Wuhan N (HR, 0.59, 95% CI, 0.37–0.93) were found to be independently associated with breakthrough infection (Table 4). No significant interactions were detected between IgG and IgA antibodies targeting S or between IgG and IgA targeting N.

**Table 4:** Summary of multivariable Cox Regression model to assess the independent association of IgG and IgA against S and N antigens with breakthrough infection

Antibody	HR	Upper CI	Lower CI	*p*-value
IgG S Omicron	0.12	0.50	0.027	**0.004**
IgA S Omicron	0.48	1.23	0.19	0.13
IgG N Wuhan	0.59	0.93	0.37	**0.025**
IgA N Wuhan	1.25	2.16	0.72	0.417

Covariates for adjustment included age, comorbidities, chronic medication, sex, smoking, and vaccination with 3 doses (ref: 2 doses), and type of primary vaccination. *N* = 359

*HR* hazard ratio

## Discussion

Our study provides valuable insight into the antibody response to SARS-CoV-2, particularly against Omicron BA.1, and its role in providing protection against Omicron breakthrough infections in the vaccinated population. Higher levels of IgG and IgA antibodies targeting the S antigens of Wuhan, Delta, and Omicron BA.1 were associated with protection against breakthrough infection in a period when Omicron variants BA.2 and BA.4/BA.5 were dominant. Prior infection with SARS-CoV-2 was found to be positively associated with antibody levels and, more importantly, with protection against breakthrough infection independently from antibody levels. Nevertheless, we also found that the longer the time elapsed since the last infection, the lower the protection, with no detectable impact of infections prior to primary vaccination and the 3rd booster. In addition, primary vaccination with BNT162b2 followed by booster vaccination with mRNA-1273 was associated with higher antibody responses than homologous mRNA-1273 vaccination for both primary and booster doses, confirming the superiority of heterologous vaccination even within mRNA vaccines [25, 26].

Despite the higher ability of Omicron variants to evade humoral immunity compared to other previous variants, our findings indicate that antibodies elicited by vaccination or hybrid immunity still played a crucial role in providing protection against breakthrough BA.2/BA.4/BA.5 infection, particularly IgG to Omicron BA.1 S. According to our results, protection is in part mediated by IgG S from Omicron variant independently of IgA, although a synergistic effect was not detected. Unfortunately, we could not distinguish or isolate the effects on protection among the IgGs to the different S antigens due to the high correlation observed among those antibodies. The fact that IgG and IgA specifically targeting the Omicron S were strongly associated with protection aligns with expectations, considering that Omicron variants were dominant during the period when breakthrough infections were assessed. These antibodies are likely to include neutralizing antibodies, which are strongly associated with protection. While we did not measure neutralizing activity, we have previously reported that neutralizing antibody response is strongly correlated (Spearman’s *ρ* ≈ 0.6 IgA, *ρ* ≈ 0.7 IgG) with anti-S binding antibodies [19]. Of note, IgG against N was also associated with protection, independently from IgG and IgA against S, probably reflecting the separate effect of recent infection from vaccination which may in addition induce other immune effector mechanisms independent from the anti-S IgG and IgA responses, such as cellular immunity.

Previous studies including ours have demonstrated that vaccination with BNT162b2 tends to result in lower antibody levels compared to mRNA-1273 [18, 27]. However, according to our results using a Luminex-based assay, heterologous vaccination regime (primary vaccination with BNT162b2 followed by a mRNA-1273 booster) appears to counteract the lower antibody levels associated with BNT162b2 primary vaccination. This finding is consistent with other studies that have shown heterologous vaccination to be associated with higher antibody levels and increased protection against infection [25, 26, 28]. However, previous studies compared homologous prime-boost vaccination with BNT162b2 to primary vaccination with BNT162b2 followed by a mRNA-1273 booster. Thus, to the best of our knowledge, this is the first study to report that primary vaccination with BNT162b2 and booster vaccination with mRNA-1273 is associated with higher antibody responses than homologous prime-boost mRNA-1273 vaccination.

Regarding the impact of the timing of infection on breakthrough infection [22–24], we did not account for the virus variant of the previous infection, but we expect timing to be highly correlated with the predominant variant at that time. While infections at pre-booster vaccination were most likely with Alpha or Delta variants (dominant in Spain in that period), infections post-booster were most likely with Delta or Omicron variants, with the likelihood of an Omicron infection being higher if the infection was more recent. Hence, it is plausible that both the time since the last infection and the particular infecting strain may impact the extent of the observed protective effect. This would be consistent with previous studies that have suggested that protection is largely determined by the variant of the prior infection rather than by the time since the last infection [29].

While our data highlights the impact of antibody levels on the risk of infection, other processes of the immune system are also crucial in the response against SARS-CoV-2. Along these lines, the association between previous infection and protection was reduced but still statistically significant after accounting for antibody levels (mediators of the protective effect), which indicates that additional mechanisms may also contribute to the overall protective effect of previous infection. Cellular immunity may hold significant importance, as T cell-based immunity has shown to be more stable over time and more conserved among variants than the antibody response [30–34].

In our study, previous infections were found to generate antibody levels that were similar or higher than those observed after booster doses. Interestingly, the combination of two vaccine doses and infection was associated with higher anti-S1 IgA and anti-RBD IgG and IgA levels compared to individuals who received three vaccine doses alone. Importantly, the time since infection and since booster vaccination were similar, suggesting that this association is not due to infections happening later than booster vaccinations. This finding aligns with previous studies indicating that hybrid immunity, achieved through a combination of vaccination and natural infection, offers enhanced protection against the disease [15, 16]. Furthermore, recent studies have demonstrated that individuals with hybrid immunity exhibit higher levels of protection against Omicron infections compared to those who have only been vaccinated [14, 29, 35].

In this study, a third vaccine dose (with respect to two doses) was associated with an increase in antibody levels against Wuhan and Delta 3 months after the booster, but not with antibodies against Omicron. In addition, we did not find an association between three-dose vaccination (with respect to two doses) and protection against breakthrough infection. This could be attributed to the lack of association between a third dose and Omicron antibody levels, together with the prevalence of more evasive Omicron strains during the breakthrough infection period [36]. These findings are consistent with multiple studies that have reported that Omicron has the ability to escape immunity and that vaccine effectiveness against this variant is lower and declines rapidly as compared to previous variants [5, 6, 30, 37]. Also, the sample size for those vaccinated with 2-doses was relatively small, which may have limited our power to detect the potential effect of a third dose.

Several limitations should be considered when interpreting our findings. Firstly, our study cohort consisted mainly of young adult women from the HCW population, which may not be fully representative of the general population. Therefore, caution should be exercised in generalizing the results to other demographics. Secondly, we lacked specific data on the viral strain responsible for breakthrough infections. However, our analysis was conducted during a time period when the vast majority of samples from Spain’s national sequencing data confirmed the prevalence of the Omicron variant (> 95%) [36]. Consequently, the potential for misclassification to other strains is likely minimal. It is worth noting that in this study, we measured serum IgA and not mucosal IgA, which may play a more relevant role in immunity against infection [38]. Another limitation lies in the assumptions made for our analysis of the association between antibody levels and protection, which rely on the uncorrelated nature of other immune mediators. Also, in this analysis, potential antibody decay after M24 was not accounted for. Furthermore, we cannot rule out the possibility of residual confounding from other behavioral and epidemiological factors when examining the associations between previous infection and protection. For instance, individuals infected earlier may have a higher number of contacts, potentially increasing their risk of reinfection compared to those without a previous infection.

## Conclusions

In conclusion, antibody levels against S antigens induced by vaccination alone or hybrid immunity, particularly IgG but also IgA against S Omicron correlate with protection against Omicron breakthrough infections. Importantly, a short time since infection in hybrid immunity and heterologous vaccination are positively associated with those protective antibody levels. However, in our study, the effect of the third dose on protection beyond 3 months was not evident. Instead, recent infection was the strongest factor associated with decreased risk of breakthrough infection, with antibody responses playing an important but partial role in mediating this protection. Therefore, our data provide valuable information for health authorities to optimize vaccination strategies and prioritize booster doses on those not recently infected to ensure sustained immune responses against evolving SARS-CoV-2 variants.

### Supplementary Information

**Additional file 1.** Correlates of protection and determinants of SARS-CoV-2 breakthroughs one year after third dose vaccination. **Supplementary Figure 1.** Comparison of antibody levels against S from Delta and Wuhan variants following hybrid immunity or vaccination alone. **Supplementary Figure 2.** Evolution and comparison of IgM levels following hybrid immunity or vaccination alone. **Supplementary Figure 3.** Evolution and comparison of antibody levels after booster vaccination according to the timing of the last SARS-CoV-2 infection in individuals vaccinated with 3 doses. **Supplementary Figure 4.** Evolution and comparison of IgM levels after booster vaccination according to the timing of the last SARS-CoV-2 infection in individuals vaccinated with 3 doses. **Supplementary Figure 5.** Comparison of antibody levels against S from Delta and Omicron variants after booster vaccination according to last SARS-CoV-2 infection in individuals vaccinated with 3 doses. **Supplementary Figure 6.** Linear regression analysis of the association of several factors with M24 IgG antibody levels in infected individuals vaccinated with 3 doses. **Supplementary Figure 7.** Evolution and comparison of antibody levels after primary vaccination with BNT162b2 or mRNA-1273 followed by booster vaccination with mRNA-1273. **Supplementary Figure 8.** Comparison of antibody levels against S from Delta and Omicron variants after primary vaccination with BNT162b2 or mRNA-1273 followed by booster vaccination with mRNA-1273. **Supplementary Figure 9.** Linear regression analysis of the association of several factors with M24 IgG levels in naïve individuals vaccinated with 3 doses. **Supplementary Figure 10.** Association of IgA levels at M24 with post-M24 breakthrough infections. **Supplementary Figure 11.** Predicted risk of breakthrough infection as a function of IgG antibody levels measured at M24. **Supplementary Figure 12.** Correlations between the different IgG, IgA and IgM antibody levels at M24. **Supplementary Figure 13.** Penalized Cox regression model to identify non-collinear antibodies highly associated with protection against breakthrough infection. **Supplementary Figure 14.** Heatmap of hierarchical clustering of study participants based on antibody levels at M24. **Supplementary Figure 15.** Directed acyclic graph (DAG) which reports our causal assumptions for generating the models to assess the total effect of a given factor on antibody levels at M24 (MFI) on naïve and infected individuals with 3 doses. **Supplementary Figure 16.** Directed acyclic graph (DAG) which reports our causal assumptions for generating the models to assess the total effect of a given factor on antibody levels at M24 (MFI) on naïve individuals with 3 doses. **Supplementary Figure 17.** Directed acyclic graph (DAG) which reports our causal assumptions for generating the models to assess the total effect of a given factor on antibody levels at M24 (MFI) on infected individuals with 3 doses. **Supplementary Figure 18.** Directed acyclic graph (DAG) which reports our causal assumptions for generating the models to assess the effect of booster vaccination (third dose) on antibody levels at M24 (MFI). **Supplementary Figure 19.** Directed acyclic graph (DAG) which reports our causal assumptions for generating the models to assess the total effect of several factors on breakthrough infection post-M24 on individuals vaccinated with 3 doses. **Supplementary Figure 20.** Directed acyclic graph (DAG) which reports our causal assumptions for generating the models to assess the total effect of several factors on breakthrough infection post-M24 on individuals vaccinated with 2 or 3 doses. **Supplementary Table 1.** Seropositivity status of study participants at M24. **Supplementary Table 2.** Summary of linear regression models to assess the association of a third dose of mRNA vaccine with IgG levels as compared to two doses. **Supplementary Table 3.** Summary of univariable Cox Regression models for the association of antibody levels with breakthrough infection in individuals vaccinated with two or three doses of mRNA vaccine. **Supplementary Table 4.** Summary of univariable Cox Regression models to assess the association between several clinicodemographic factors with breakthrough infection in individuals vaccinated with two or three doses of mRNA vaccine.

## Data Availability

The datasets generated and/or analyzed during the current study are available in CORA repository, (10.34810/data1122). The raw identifying data are protected and are not available due to data privacy laws. Code used in the analysis is available at CORA repository (10.34810/data1122).

## Abbreviations

AE: Adverse event
DAG: Directed acyclic graph
GAM: Generalized additive model
HCW: Health care workers
HR: Hazard ratio
N: Nucleocapsid
RBD: Receptor-binding domain
rRT-PCR: Real-time reverse transcription-polymerase chain reaction
S: Spike
