# Supplementary material for: Correlates of protection and determinants of SARS-CoV-2 breakthrough infections 1 year after third dose vaccination
Source: BMC Med. 2024 Mar 8;22:103. doi: 10.1186/s12916-024-03304-3 (PMC10921636; doi:10.1186/s12916-024-03304-3)
Supplement: Supplementary file 1 — Additional file 1. Correlates of protection and determinants of SARS-CoV-2 breakthroughs one year after third dose vaccination. Supplementary Figure 1. Comparison of antibody levels against S from Delta and Wuhan variants following hybrid immunity or vaccination alone. Supplementary Figure 2. Evolution and comparison of IgM levels following hybrid immunity or vaccination alone. Supplementary Figure 3. Evolution and comparison of antibody levels after booster vaccination according to the timing of the last SARS-CoV-2 infection in individuals vaccinated with 3 doses. Supplementary Figure 4. Evolution and comparison of IgM levels after booster vaccination according to the timing of the last SARS-CoV-2 infection in individuals vaccinated with 3 doses. Supplementary Figure 5. Comparison of antibody levels against S from Delta and Omicron variants after booster vaccination according to last SARS-CoV-2 infection in individuals vaccinated with 3 doses. Supplementary Figure 6. Linear regression analysis of the association of several factors with M24 IgG antibody levels in infected individuals vaccinated with 3 doses. Supplementary Figure 7. Evolution and comparison of antibody levels after primary vaccination with BNT162b2 or mRNA-1273 followed by booster vaccination with mRNA-1273. Supplementary Figure 8. Comparison of antibody levels against S from Delta and Omicron variants after primary vaccination with BNT162b2 or mRNA-1273 followed by booster vaccination with mRNA-1273. Supplementary Figure 9. Linear regression analysis of the association of several factors with M24 IgG levels in naïve individuals vaccinated with 3 doses. Supplementary Figure 10. Association of IgA levels at M24 with post-M24 breakthrough infections. Supplementary Figure 11. Predicted risk of breakthrough infection as a function of IgG antibody levels measured at M24. Supplementary Figure 12. Correlations between the different IgG, IgA and IgM antibody levels at M24. Supplementary Figure 13. Penalized [file 12916_2024_3304_MOESM1_ESM.docx]

**Figure S1.** Comparison of antibody levels against S from Delta and Wuhan variants following hybrid immunity or vaccination alone. Antibody levels (MFI) at M24 are compared according to exposure (2 doses followed by infection (n = 22), 2 doses without infection (n = 13), and 3 doses without infection (n = 129)). The center line on each box corresponds to the median MFI, the lower and upper hinges correspond to the first and third quartiles, and the whiskers extend from the hinge to the highest or lowest value within 1·5 × IQR of the respective hinge. Grey dashed line depicts the positivity cutoff for the given antibody. Statistical significance was tested by Wilcoxon rank-sum test and adjusted for multiple comparisons using Benjamini-Hochberg method.

**Figure S2.** Evolution and comparison of IgM levels following hybrid immunity or vaccination alone. Plots show IgM levels against the receptor-binding domain (RBD) of the SARS-CoV-2 Spike glycoprotein (S), S, its subunits S1 and S2, and against the nucleocapsid (N). **a**. Antibody levels (MFI/cutoff) at M12 and M24 time points in individuals vaccinated with 2 doses followed by infection (n = 22), 2 doses without infection (n = 13), and 3 doses without infection (n = 129). The data shown are geometric mean in each group ± geometric SD. Statistical significance was tested by Wilcoxon signed-rank test and adjusted for multiple comparisons using Benjamini-Hochberg method. **b**. Comparison of antibody levels (MFI) at M24 according to exposure. The center line on each box corresponds to the median MFI, the lower and upper hinges correspond to the first and third quartiles, and the whiskers extend from the hinge to the highest or lowest value within 1·5 × IQR of the respective hinge. Grey dashed line depicts the positivity cutoff for the given antibody. Statistical significance was tested by Wilcoxon rank-sum test and adjusted for multiple comparisons using Benjamini-Hochberg method.

**Figure S3.** Evolution and comparison of antibody levels after booster vaccination according to the timing of the last SARS-CoV-2 infection in individuals vaccinated with 3 doses. Plots show IgA and IgG levels against the receptor-binding domain (RBD) of the SARS-CoV-2 Spike glycoprotein (S), S, its subunits S1 and S2, and against the nucleocapsid (N). **a**. Scheme illustrating the order and approximate time scale of vaccination and infection for each group. The syringe indicates a vaccine dose, the virus particle indicates an infection with SARS-CoV-2, and the blood droplet indicates plasma collection. **b**. Antibody levels (MFI/cutoff) at M12 and M24 time points in naïve (n = 129), infected pre-primary vaccination (n = 40), infected post-primary pre-booster vaccination (n = 14), and infected post-booster vaccination (n = 71) individuals. The data shown are geometric mean in each group ± geometric SD. Statistical significance was tested by Wilcoxon signed-rank test and adjusted for multiple comparisons using Benjamini-Hochberg method. **c**. Comparison of antibody levels (MFI) at M24 according to last infection period. The center line on each box corresponds to the median MFI, the lower and upper hinges correspond to the first and third quartiles, and the whiskers extend from the hinge to the highest or lowest value within 1·5 × IQR of the respective hinge. Grey dashed line depicts the positivity cutoff for the given antibody. Statistical significance was tested by Wilcoxon rank-sum test and adjusted for multiple comparisons using Benjamini-Hochberg method.

**Figure S4.** Evolution and comparison of IgM levels after booster vaccination according to the timing of the last SARS-CoV-2 infection in individuals vaccinated with 3 doses. Plots show IgM levels against the receptor-binding domain (RBD) of the SARS-CoV-2 Spike glycoprotein (S), S, its subunits S1 and S2, and against the nucleocapsid (N). **a**. Antibody levels (MFI/cutoff) at M12 and M24 time points in naïve (n = 129), infected pre-primary vaccination (n = 40), infected post-primary pre-booster vaccination (n = 14), and infected post-booster vaccination (n = 71) individuals. The data shown are geometric mean in each group ± geometric SD. Statistical significance was tested by Wilcoxon signed-rank test and adjusted for multiple comparisons using Benjamini-Hochberg method. **b**. Comparison of antibody levels (MFI) at M24 according to last infection period. The center line on each box corresponds to the median MFI, the lower and upper hinges correspond to the first and third quartiles, and the whiskers extend from the hinge to the highest or lowest value within 1·5 × IQR of the respective hinge. Grey dashed line depicts the positivity cutoff for the given antibody. Statistical significance was tested by Wilcoxon rank-sum test and adjusted for multiple comparisons using Benjamini-Hochberg method.

**Figure S5.** Comparison of antibody levels against S from Delta and Omicron variants after booster vaccination according to last SARS-CoV-2 infection in individuals vaccinated with 3 doses. Antibody levels (MFI) at M24 are compared according to last infection period (naïve (n = 129), infected pre-primary vaccination (n = 40), infected post-primary pre-booster vaccination (n = 14), and infected post-booster vaccination (n = 71)). The center line on each box corresponds to the median MFI, the lower and upper hinges correspond to the first and third quartiles, and the whiskers extend from the hinge to the highest or lowest value within 1·5 × IQR of the respective hinge. Grey dashed line depicts the positivity cutoff for the given antibody. Statistical significance was tested by Wilcoxon rank-sum test and adjusted for multiple comparisons using Benjamini-Hochberg method.

**Figure S6.** Linear regression analysis of the association of several factors with M24 IgG antibody levels in infected individuals vaccinated with 3 doses. Forest plots show the association of age, comorbidities, primary vaccination type, sex, smoking, systemic adverse effects (AEs), time since dose 3, and time since last infection with IgG antibody levels at M24. The estimates (dots) and 95% CI were calculated using linear regression models. Beta (β) and CI values have been transformed to a percentage for an easier interpretation. The color of the dots represents the P value after adjustment for multiple testing by Benjamini-Hochberg, where black represents < 0.001, dark grey < 0.01, light grey < 0.05, and white non-significant. ^a^ Non-adjusted variable. N = 158. ^b^ Covariates for adjustment included age, sex and smoking. N = 158. ^c^ Covariates for adjustment included age and sex. N = 158. ^d^ Covariates for adjustment included age, comorbidities, chronic medication, sex, smoking, primary vaccination type. N = 158. ^e^ Non-adjusted variable. N = 80.

**Figure S7.** Evolution and comparison of antibody levels after primary vaccination with BNT162b2 or mRNA-1273 followed by booster vaccination with mRNA-1273. Plots show IgA and IgG levels against the receptor-binding domain (RBD) of the SARS-CoV-2 Spike glycoprotein (S), S, its subunits S1 and S2, and against the nucleocapsid (N). **a**. Antibody levels (MFI/cutoff) at M12 and M24 time points in individuals vaccinated with 2 doses of BNT162b2 (n = 225) or mRNA-1273 (n = 60) followed by booster vaccination with mRNA-1273. The data shown are geometric mean in each group ± geometric SD. Statistical significance was tested by Wilcoxon signed-rank test and adjusted for multiple comparisons using Benjamini-Hochberg method. **b**. Comparison of antibody levels (MFI) at M24 according to primary vaccination type (BNT162b2 (n = 225) or mRNA-1273 (n = 60)). The center line on each box corresponds to the median MFI, the lower and upper hinges correspond to the first and third quartiles, and the whiskers extend from the hinge to the highest or lowest value within 1·5 × IQR of the respective hinge. Grey dashed line depicts the positivity cutoff for the given antibody. Statistical significance was tested by Wilcoxon rank-sum test and adjusted for multiple comparisons using Benjamini-Hochberg method.

**Figure S8**. Comparison of antibody levels against S from Delta and Omicron variants after primary vaccination with BNT162b2 or mRNA-1273 followed by booster vaccination with mRNA-1273. Antibody levels (MFI) at M24 are compared according to primary vaccination type (BNT162b2 (n = 225) or mRNA-1273 (n = 60)). The center line on each box corresponds to the median MFI, the lower and upper hinges correspond to the first and third quartiles, and the whiskers extend from the hinge to the highest or lowest value within 1·5 × IQR of the respective hinge. Grey dashed line depicts the positivity cutoff for the given antibody. Statistical significance was tested by Wilcoxon rank-sum test and adjusted for multiple comparisons using Benjamini-Hochberg method.

**Figure S9.** Linear regression analysis of the association of several factors with M24 IgG levels in naïve individuals vaccinated with 3 doses. Forest plots show the association of age, comorbidities, primary vaccination type, sex, smoking, systemic adverse effects (AEs) after each dose, time since dose 2, and time since dose 3 with IgG levels at M24. The estimates (dots) and 95% CI were calculated using linear regression models. Beta (β) and CI values have been transformed to a percentage for an easier interpretation. The color of the dots represents the P value after adjustment for multiple testing by Benjamini-Hochberg, where black represents < 0.001, dark grey < 0.01, light grey < 0.05, and white non-significant. ^a^ Non-adjusted variable. ^b^ Covariates for adjustment included age, sex and smoking. ^c^ Covariates for adjustment included age and sex. ^d^ Covariates for adjustment included age, comorbidities, chronic medication, sex, smoking, primary vaccination type. N = 129.

**Figure S10.** Association of IgA levels at M24 with post-M24 breakthrough infections. **a**. IgA levels (MFI) at M24 against the receptor-binding domain (RBD) of Wuhan SARS-CoV-2 Spike glycoprotein (S), S, its subunits S1 and S2, and against the nucleocapsid (N), in breakthrough infected (n = 95) and non-breakthrough infected (n = 274) individuals. **b**. IgG levels (MFI) at M24 against Delta and Omicron S. The center line on each box corresponds to the median MFI, the lower and upper hinges correspond to the first and third quartiles, and the whiskers extend from the hinge to the highest or lowest value within 1·5 × IQR of the respective hinge. Grey dashed line depicts the positivity cutoff for the given antibody. Statistical significance was tested by Wilcoxon rank-sum test and adjusted for multiple comparisons using Benjamini-Hochberg method.

**Figure S11.** Predicted risk of breakthrough infection as a function of IgG antibody levels measured at M24. Blue shaded areas represent the density distribution for each antibody. Purple lines show the risk of infection. Purple shaded areas represent the 95% CIs for the risk.

**Figure S12.** Correlations between the different IgG, IgA and IgM antibody levels at M24. Pearson correlation R values are shown in the heatmap.

**Figure S13**. Penalized Cox regression model to identify non-collinear antibodies highly associated with protection against breakthrough infection. **a**. Coefficient profile for antibody variables in the LASSO Cox regression model. lambda.min (the value of the tuning parameter corresponding to the minimum mean cross-validation error) and lambda.1se (the largest value of λ such that error is within 1 standard error of the minimum) are indicated by the vertical red and blue lines, respectively. **b**. Lambda (λ) parameter selection in the model. The partial likelihood deviance is plotted against log(λ), in which vertical lines are shown at the lambda.min (first line) and lambda.1se (second line).

**Figure S14.** Heatmap of hierarchical clustering of study participants based on antibody levels at M24. Columns represent antibody levels, and rows represent participants. Antibody levels are shown on a color scale from blue to red. Individual post-M24 breakthrough infection status is overlaid on the left of the heatmap (0 – non-infected, 1 – breakthrough infection).

**Figure S15**. Directed acyclic graph (DAG) which reports our causal assumptions for generating the models to assess the total effect of a given factor on antibody levels at M24 (MFI) on naïve and infected individuals with 3 doses. Figure created using <http://www.dagitty.net/dags.html>

Legend text: Primary vaccination type includes BNT162b2 and mRNA-1273; MFI M24 = levels of anti-SARS-CoV-2 antibodies at M24, AE = Adverse effects.

**Figure S16.** Directed acyclic graph (DAG) which reports our causal assumptions for generating the models to assess the total effect of a given factor on antibody levels at M24 (MFI) on naïve individuals with 3 doses. Figure created using <http://www.dagitty.net/dags.html>

Legend text: MFI M24 = levels of anti-SARS-CoV-2 antibodies at M24, AE = Adverse effects.

**Figure S17**. Directed acyclic graph (DAG) which reports our causal assumptions for generating the models to assess the total effect of a given factor on antibody levels at M24 (MFI) on infected individuals with 3 doses. Figure created using <http://www.dagitty.net/dags.html>

Legend text: Primary vaccination type includes BNT162b2 and mRNA-1273; MFI M24 = levels of anti-SARS-CoV-2 antibodies at M24, AE = Adverse effects.

**Figure S18.** Directed acyclic graph (DAG) which reports our causal assumptions for generating the models to assess the effect of booster vaccination (third dose) on antibody levels at M24 (MFI). Figure created using <http://www.dagitty.net/dags.html>

Legend text: MFI M24 = levels of anti-SARS-CoV-2 antibodies at M24, AE = Adverse effects.

.

**Figure S19.** Directed acyclic graph (DAG) which reports our causal assumptions for generating the models to assess the total effect of several factors on breakthrough infection post-M24 on individuals vaccinated with 3 doses. Figure created using <http://www.dagitty.net/dags.html>

Legend text: Primary vaccination type includes BNT162b2 and mRNA-1273; MFI M24 = levels of anti-SARS-CoV-2 antibodies at M24.

**Figure S20.** Directed acyclic graph (DAG) which reports our causal assumptions for generating the models to assess the total effect of several factors on breakthrough infection post-M24 on individuals vaccinated with 2 or 3 doses. Figure created using <http://www.dagitty.net/dags.html>

Legend text: Ab M24 = levels of anti-SARS-CoV-2 antibodies at M24.

**Table S2**. Seropositivity status of study participants at M24.

| Variant | Isotype | Antigen | Seronegative | | Indeterminate | | Seropositive | |
| --- | --- | --- | --- | --- | --- | --- | --- | --- |
|  |  |  | n | % | n | % | n | % |
| Wuhan | IgA | S | 6 | 2 | 1 | 0 | 386 | 98 |
|  |  | N | 332 | 84 | 35 | 9 | 36 | 9 |
|  | IgG | S | 0 | 0 | 0 | 0 | 393 | 100 |
|  |  | N | 237 | 60 | 90 | 23 | 66 | 17 |
|  | IgM | S | 320 | 81 | 37 | 9 | 36 | 9 |
|  |  | N | 379 | 96 | 11 | 3 | 3 | 1 |
| Omicron | IgA | S | 109 | 28 | 94 | 24 | 186 | 47 |
|  | IgG | S | 41 | 10 | 352 | 90 | 0 | 0 |
|  | IgM | S | 390 | 99 | 2 | 1 | 0 | 0 |
| Delta | IgA | S | 41 | 10 | 132 | 34 | 216 | 55 |
|  | IgG | S | 3 | 1 | 47 | 12 | 343 | 87 |
|  | IgM | S | 376 | 96 | 15 | 4 | 2 | 1 |

**Table S3**. Summary of linear regression models to assess the association of a third dose of mRNA vaccine with IgG levels as compared to two doses.

| Antigen | Variant | β (%) | Upper 95% CI | Lower 95% CI | P-value |
| --- | --- | --- | --- | --- | --- |
| S | Wuhan | 26.33 | 44.74 | 10.26 | **0.0008** |
| S1 | Wuhan | 24.39 | 43.39 | 7.91 | **0.0027** |
| S2 | Wuhan | 8.94 | 30.01 | -8.71 | 0.3415 |
| N | Wuhan | -37.37 | -18.16 | -52.06 | **0.0006** |
| RBD | Wuhan | 25.42 | 48.03 | 6.26 | **0.0075** |
| S | Delta | 25.32 | 44.13 | 8.95 | **0.0016** |
| S | Omicron | 8.14 | 17.95 | -0.85 | 0.0707 |

Covariates for adjustment included age, comorbidities, chronic medication, sex, smoking, and infection. N = 366. Beta (β) and CI values have been transformed to a percentage for an easier interpretation.

**Table S4**. Summary of univariable Cox Regression models for the association of antibody levels with breakthrough infection in individuals vaccinated with two or three doses of mRNA vaccine.

| Isotype | Antigen | Variant | HR | Upper CI | Lower CI | P-value |
| --- | --- | --- | --- | --- | --- | --- |
| IgA | N | Wuhan | 0.36 | 0.73 | 0.18 | **0.0049** |
| IgA | RBD | Wuhan | 0.45 | 0.64 | 0.32 | **< 0.0001** |
| IgA | S | Wuhan | 0.44 | 0.64 | 0.31 | **< 0.0001** |
| IgA | S1 | Wuhan | 0.46 | 0.65 | 0.32 | **< 0.0001** |
| IgA | S2 | Wuhan | 0.49 | 0.73 | 0.33 | **0.0005** |
| IgG | N | Wuhan | 0.39 | 0.60 | 0.26 | **< 0.0001** |
| IgG | RBD | Wuhan | 0.29 | 0.47 | 0.18 | **< 0.0001** |
| IgG | S | Wuhan | 0.26 | 0.48 | 0.14 | **< 0.0001** |
| IgG | S1 | Wuhan | 0.27 | 0.47 | 0.15 | **< 0.0001** |
| IgG | S2 | Wuhan | 0.32 | 0.54 | 0.19 | **< 0.0001** |
| IgA | S | Delta | 0.47 | 0.71 | 0.31 | **0.0003** |
| IgG | S | Delta | 0.29 | 0.53 | 0.16 | **0.0001** |
| IgA | S | Omicron | 0.10 | 0.24 | 0.04 | **< 0.0001** |
| IgG | S | Omicron | 0.05 | 0.15 | 0.02 | **< 0.0001** |

N = 277. HR, hazard ratio.

**Table S5**. Summary of univariable Cox Regression models to assess the association between several clinico-demographic factors with breakthrough infection in individuals vaccinated with two or three doses of mRNA vaccine.

| Factor | HR | Upper CI | | Lower CI | | P-value |
| --- | --- | --- | --- | --- | --- | --- |
| Age^a^ | 0.996 | | 1.013 | | 0.980 | 0.6589 |
| Comorbidites^a^ | 0.867 | | 1.327 | | 0.566 | 0.5100 |
| Infection (ref: no infection) ^b^ | 0.526 | | 0.768 | | 0.360 | **0.0004** |
| Sex (ref: male) ^a^ | 1.465 | | 2.322 | | 0.924 | 0.1044 |
| Smoker^c^ | 0.777 | | 1.276 | | 0.474 | 0.3190 |
| Time since last dose (days) ^a^ | 0.998 | | 1.001 | | 0.996 | 0.1360 |
| Vaccination with 3 doses (ref: 2 doses) ^a^ | 1.963 | | 3.346 | | 1.151 | **0.0132** |

^a^ N = 369, ^b^ N = 366, ^c^ N = 368. HR, hazard ratio.
